# Validating robotic couch isocentricity with 3D surface imaging

**DOI:** 10.1002/acm2.12939

**Published:** 2020-06-15

**Authors:** Omar El‐Sherif, Nicholas B. Remmes, Jon J. Kruse

**Affiliations:** ^1^ Mayo Clinic Department of Radiation Oncology Rochester MN USA

**Keywords:** 3D surface imaging, AlignRT, patient positioning, quality assurance, VisionRT

## Abstract

**Background:**

A proton therapy system with 190° gantries uses robotic couch rotations to change the treatment beam laterality. Couch rotations are typically validated clinically with post‐rotation radiographic imaging.

**Aims:**

This study assesses the specificity and sensitivity of a commercial 3D surface imaging system, AlignRT (Vision RT, London UK) for validating couch rotations.

**Materials & Methods:**

In clinical operation, a reference surface image of the patient is acquired after radiographic setup with couch at 270°, perpendicular to the gantry axis of rotation. The couch is then rotated ±90° to a typical treatment angle, and AlignRT reports a 3D displacement vector. Patient motion, changes in patient surface, non‐coincidence between AlignRT and couch isocenter, and mechanical couch run‐out all contribute to the 3D vector magnitude. To assess AlignRT sensitivity in detecting couch run‐out, volunteers were positioned orthogonal to the proton gantry and reference surface images were captured without x‐ray localization. Subjects were repeatedly rotated ±90⁰ to typical treatment angles and displacement vectors were recorded. Additionally, measurements were performed in which intentional translations of 2, 4, 6, and 8 mm were combined with the intended isocentric rotations. Data sets were collected using a phantom; subjects with a thoracic isocenter and no immobilization; and subjects with a cranial isocenter and thermoplastic immobilization. A total of 300 rotations were measured.

**Results:**

During isocentric rotations, the mean AlignRT displacement vectors for the phantom, immobilized, and non‐immobilized volunteers were 0.1 ± 0.1 mm, 0.8 ± 0.1 mm, and 1.1 ± 0.2 mm respectively. 95% of the AlignRT measurements for the immobilized and non‐immobilized subjects were within 1 mm and 2 mm of the actual displacement respectively.

**Discussion:**

After characterizing the accuracy using phantoms and volunteers, we have shown that a three‐pod surface imaging system can be used to identify gross non‐isocentric patient rotations. Significant positional deviations, either due to improper couch rotation or patient motion, should be followed by radiographic imaging and repositioning.

**Conculsion:**

AlignRT can be used to verify patient positioning following couch rotations that are applied after the initial x‐ray guided patient setup. Using a three‐pod AlignRt system, positional deviations exceeding 4 mm were flagged with sensitivity and specificity of 90% and 100% respectively.

## 1 INTRODUCTION

**Fig. 1:**
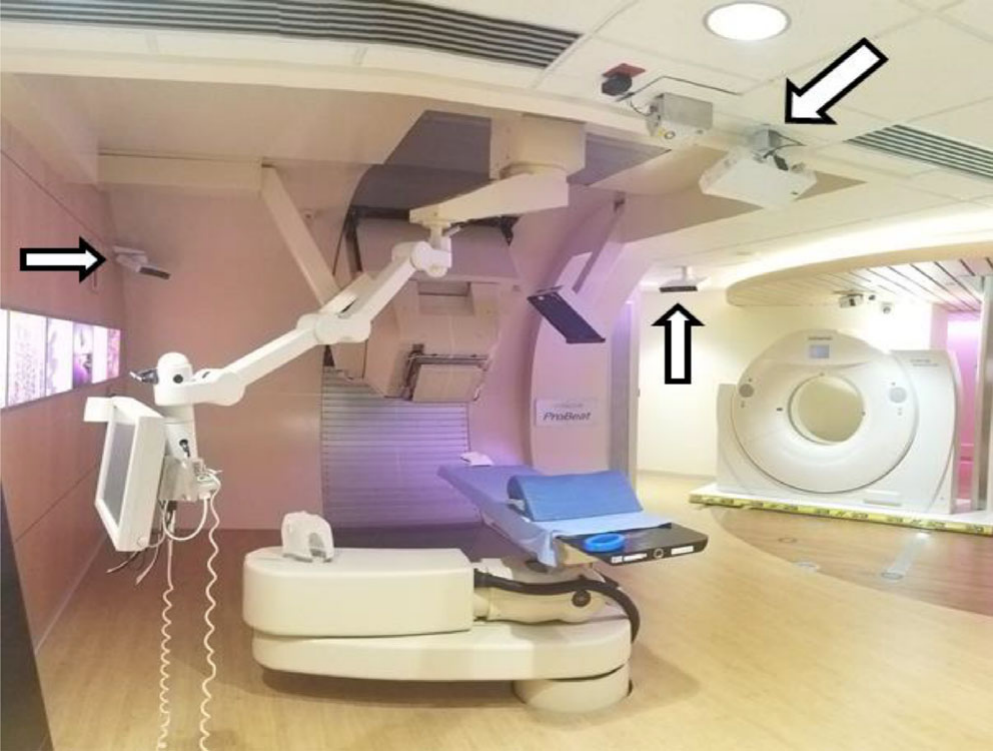
Photograph of three ceiling mounted camera pods (white arrows) of the surface image guidance system within the half‐gantry proton treatment room.

The Mayo Clinic proton therapy facility features four treatment rooms with half gantries (~190° range of motion), as shown in Fig. 1. The half gantry design often necessitates one or more rotations of the treatment couch to achieve the desired beam angles during a single radiotherapy session. Couch mechanical isocentricity is maintained to <1 mm runout during monthly quality assurance tests as per previously published recommendations.^1^, ^2^ Unlike a traditional pedestal‐style linac couch, however, isocentric rotations of a robotic couch require precise coordination of seven independent motors (the ‘shoulder”, the “elbow” and the “wrist,” as well as three rotational motors in the wrist and a vertical axis drive). Additionally, force sensors in the robotic couch detect the weight and location of center of gravity of the load on the couch surface since mechanical sag of the carbon fiber couch top must be accounted for to achieve mechanical isocentricity.^3^

As in photon radiotherapy, x‐ray image guidance systems are used for pretreatment patient localization. Patients are typically setup with the couch longitudinal axis perpendicular to the proton gantry axis of rotation, as shown in Fig. 1 (Couch angle = 270°). X‐ray panels suspended from the ceiling provide posterior‐lateral 45° oblique images for patient localization. The patient is commonly rotated 90° (to couch angle 0° or 180°) to achieve treatment beam angles. Initial localization can be more difficult with the couch in treatment orientation, as superior–inferior imaging angles limit the ability to resolve features such as intervertebral spaces. Because of the complex nature of a robotic isocentric rotation, patients are imaged with the x‐ray system after rotation to validate isocentricity of the operation. However, obtaining radiographs after every couch rotation slows the treatment process and increases the radiation exposure to the patient's healthy tissues. In the first several years of operation of the Mayo Clinic proton therapy facility, thousands of postrotation radiographs were acquired to ensure that deviations in the setup were below action limits defined by the robustness of our treatment plans. The fraction which showed an actionable deviation (often ≥ 3 mm) was small (<1 in 50 patients).

In the interest of increasing treatment efficiency and reducing x‐ray dose to the patient, an argument could be made for suspending procedural radiographic imaging after each couch rotation. Five years of operation and a continuous quality assurance program have demonstrated consistently excellent performance of the robotic positioner. However, owing to the complexities of robotic rotations, it is prudent to employ some type of “second check” on the robotic couch to guard against spurious errors well in excess of normal treatment tolerances. Optical three‐dimensional (3D) surface‐based imaging systems^4^, ^5^ could potentially serve as that second check. These optical systems have quick response times compared to x‐ray imaging and do not expose the patient to ionizing radiation. The accuracy of a commercially available optical 3D surface imaging system, AlignRT (VisionRT Ltd., London UK) has been shown by several studies to be within **1 mm.** ^5^, ^6^

Here we investigate sensitivity and specificity of a surface imaging system for identifying excessive mechanical runout in a robotic couch rotation. While the intrinsic accuracy of AlignRT has been shown to be on the order of 1 mm, the postrotation deviation of a patient surface image will contain contributions from patient motion due to couch acceleration, respiratory motion, unintended voluntary motion of the patient, and finally, mechanical couch runout. It is not the goal of this work to assess the appropriateness of using the surface imaging alone for monitoring patient position during extremely precise treatments such as stereotactic radiosurgery. Rather this study attempts to determine whether the system can suitably identify spurious mechanical errors of a robotic couch.

## 2 MATERIALS AND METHODS

**Fig. 2:**
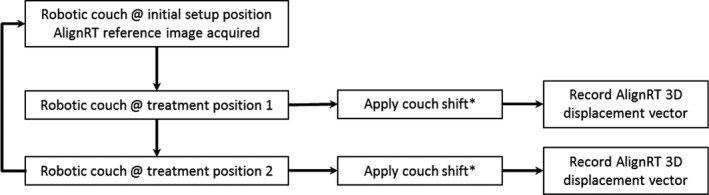
Schematic of the methodology. Treatment positions 1 and 2 consist of couch rotations that position the phantom/volunteers orthogonal to the initial setup position. ^*^Lateral, longitudinal, vertical, couch shifts of varying magnitude from isocenter are applied individually.

A phantom and healthy volunteers were used to determine the sensitivity of a three‐pod AlignRT system (see Fig. 1) in detecting clinically significant patient misalignment after couch rotations. Intrinsic isocentricity of the robotic couch and the AlignRT system were determined with a rigid phantom on the couch top using the procedure outlined in Fig. 2. A reference surface image of the phantom was acquired at the normal setup orientation (couch angle = 270°) and the couch was rotated ± 90° and then back to 270°. A treatment verification surface image was acquired after every couch rotation and the deviation in X, Y, and Z of the surface image, as well as a 3D vector length, were reported by the AlignRT system. This process was repeated with a newly acquired reference surface back at 270° after each set of ±90° rotations.

Following the baseline testing, clinical operation was simulated with healthy volunteers. Thoracic treatments were simulated with treatment isocenter in the subject’s chest and no immobilization besides a knee cushion for comfort. Head and neck treatments were simulated with treatment isocenter in the subject’s head and the volunteers immobilized under a five‐point thermoplastic mask (Orfit®, Jericho, New York, USA).

AlignRT reference surface images were acquired with the couch at setup position (couch angle 270°). The couch was rotated isocentrically ±90° to one of two common treatment positions, and a verification surface image was acquired. The deviation in X, Y, and Z of the surface image, as well as a 3D vector length, were reported by the AlignRT system. This process was repeated with a newly acquired reference surface back at 270° after each set of ±90° rotations.

Next, mechanical deviations of the couch were simulated. The couch was rotated ±90° from the setup position as before, but translations were added to the motion as well. Translations of 2, 4, 6, and 8 mm in X, Y, and Z directions were meant to mimic unintentional runout accompanying an isocentric rotation. As in the case of isocentric rotations, verification surface images were acquired after every motion, and the reported deviations were compared to the known couch runouts. Mean, standard deviation, and max were derived from a total of 30° couch rotations. The mean was defined as the average readout of the magnitudes from the AlignRT user interface (UI). The standard deviation was defined as the standard deviation calculated from the tallied magnitude readouts from the AlignRT UI. The max statistic was defined as the largest of the tallied magnitude readout from the UI.

Two action levels were tested and true and false positive rates (TP and FP), and true and false negative rates (TN and FN) of the system's ability to detect non‐isocentric couch rotations were estimated and tabulated into an error matrix. The sensitivity and specificity of the system’s ability to detect couch rotations with deviations greater than a predetermined “action level” were estimated. The sensitivity and specificity was evaluated for action levels of 2 and 4 mm. The TP was defined as the number of times the surface imaging system correctly identified rotational deviations that were greater than the action level. The FP was defined as the frequency at which the surface imaging system incorrectly flagged rotational deviations that were in fact below the set action level. The TN was defined as the number of times the surface imaging system correctly identified that the rotational deviations were within the set action level. FN was defined as the frequency at which the surface imaging system did not flag rotational deviations that were in fact greater than the set action level. The sensitivity was defined as the ratio of TP to (TP + FN). The specificity was defined as the ratio of TN to (TN + FP).

## 3 RESULTS

A statistical summary of the AlignRT reported deviations from a true isocentric couch rotation are presented in Table 1. The phantom measurements following consecutive couch rotations without preprogrammed runouts from isocenter resulted in a mean displacement of 0.01 cm ± 0.01 between the expected and captured phantom surface positions. The maximum observed deviation from the repeated measurements was 0.03 cm. These values represent an estimate of the baseline aggregated uncertainty of the accuracy of the couch movement and the accuracy of the surface image guidance system.

**Table 1:** Statistical summary of the AlignRT mean and max magnitude readouts of a phantom.

	Isocenter shift magnitude				
	0 cm	0.2 cm	0.4 cm	0.6 cm	0.8 cm
Phantom					
Mean	0.01	0.20	0.40	0.60	0.80
SD	0.01	0.01	0.01	0.01	0.01
Max (∆)	0.03 (0.03)	0.21 (0.01)	0.42 (0.02)	0.62 (0.02)	0.82 (0.02)

(∆) represents the difference between the maximum readout and the true offset.

The accuracy of the surface guidance system was assessed using a rigid phantom and predetermined translational offsets from isocenter. A mean error of 0.01 cm was found between the centroid of the phantom surface image and the expected centroid position using the positional coordinates of the robotic couch. The 95% confidence interval of the error was below 0.02 cm.

**Fig. 3:**
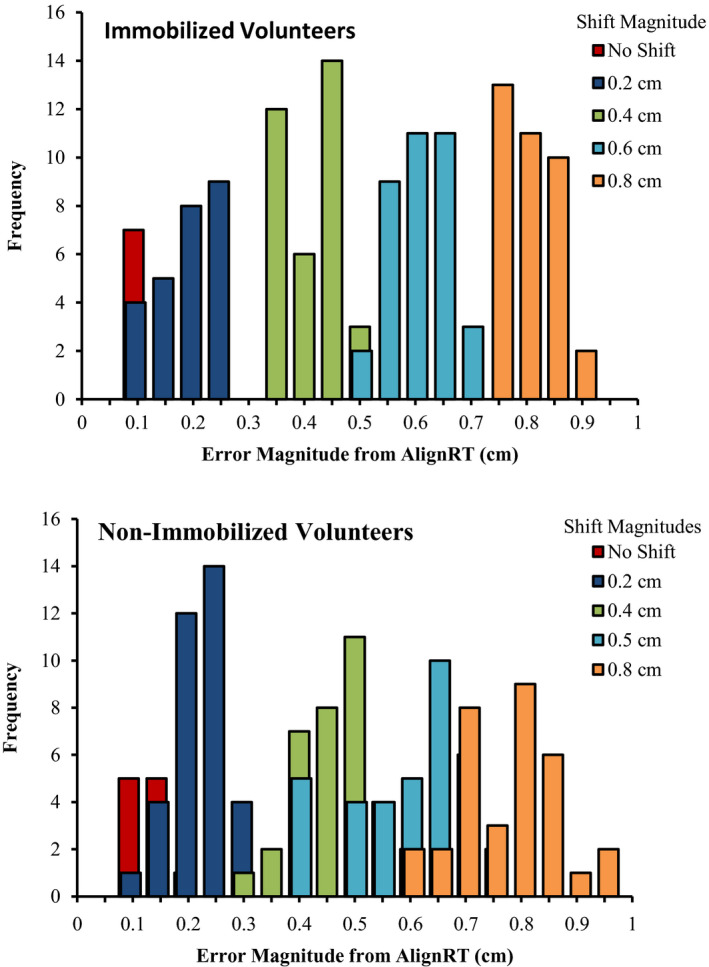
A distribution of the reported deviation magnitude for AlignRT following 0, 0.2, 0.4, 0.6, and 0.8 cm shifts from isocenter, in conjunction with a 90° rotation. Upper and lower panels represent the results from immobilized and nonimmobilized volunteers respectively.

Volunteers were used to simulate a potential clinical utilization of the surface guidance system which detects patient motion in addition to mechanical runout. A statistical summary of the AlignRT reported deviations from a true isocentric couch rotation for the volunteers are presented in Table 2. The consecutive couch rotations without preprogrammed offsets from isocenter for the nonimmobilized and immobilized volunteers resulted in a mean observed runout of 0.11 and 0.08 cm respectively. The maximum observed deviation was 0.17 and 0.10 cm for the nonimmobilized and immobilized volunteers respectively. The 95% confidence interval of the patient displacements from isocenter reported by the surface imaging system was 0.06–0.10 cm for the immobilized volunteer (0.07–0.15 cm for the nonimmobilized volunteer). The mean absolute errors between the centroid of the volunteer’s surface image and the expected centroid position were 0.05 and 0.07 cm for immobilized and nonimmobilized volunteers respectively. The distributions of the magnitude readouts from the VisionRT user interface for each predetermined translational offset from isocenter are shown in Fig. 3.

**Table 2:** Statistical summary of the AlignRT mean and max magnitude readouts of volunteers.

	Isocenter shift magnitude				
	0 cm	0.2 cm	0.4 cm	0.6 cm	0.8 cm
Non‐immobilized					
Mean	0.11	0.20	0.44	0.58	0.76
SD	0.02	0.05	0.07	0.10	0.11
Max (∆)	0.17 (0.17)	0.30 (0.10)	0.59 (0.19)	0.73 (0.13)	1.06 (0.26)
Immobilized					
Mean	0.08	0.17	0.39	0.58	0.78
SD	0.01	0.05	0.06	0.05	0.05
Max (∆)	0.1 (0.1)	0.25 (0.05)	0.50 (0.10)	0.67 (0.07)	0.87 (0.07)

(∆) represents the difference between the maximum readout and the true offset.

VisionRT’s sensitivity and specificity in detecting couch rotations with deviations greater than a predetermined “action level” were estimated. The true/false positives and true/false negatives are shown for action levels of 2 and 4 mm in Tables 3 and 4 respectively. Setting an action level of 2 mm resulted in a runout detection sensitivity and specificity of 88% and 100% respectively. Setting an action level of 4 mm resulted in a runout detection sensitivity and specificity of 90% and 100% respectively.

**Table 3:** Represents the number of true/false positives and true/false negatives for detecting couch runouts ≥2 mm using a 2 mm action level on the AlignRT displacement vector.

True runout	Number of AlignRT displacements		Total
	≥2 mm action level	<2 mm action level	
≥2 mm	248	32	280
<2 mm	0	20	20
Total	248	52	300

**Table 4:** Represents the number of true/false positives and true/false negatives for detecting couch runouts ≥4 mm using a 4 mm action level on the AlignRT displacement vector.

True runout	Number of AlignRT displacements		Total
	≥4 mm action level	<4 mm action level	
≥4 mm	186	20	206
<4 mm	0	94	94
Total	186	114	300

## 4 DISCUSSION

A half gantry proton system requires many couch rotations to achieve the desired beam angles. The complex nature of a robotic rotation warrants some form of positional verification. Although verification can be achieved with x rays, the use of optical surface imaging provides some clinical advantages. The use of surface imaging for patient position verification can improve patient throughput and reduce x‐ray dose to the patient. Here a subset of our experience in commissioning the use of a surface imaging system for patient position verification is presented. We have shown that surface imaging can be used to “flag” patient positional deviations ≥4 mm that may arise from nonisocentric treatment couch rotations and/or intrafraction patient motion.

Previous studies have assessed the accuracy of surface image guidance following treatment couch translations, however the range of tested rotations is less extensive and does not cover the typical range of couch angles seen in a half gantry proton clinic.^7^, ^8^, ^9^, ^10^, ^11^ To the best of our knowledge, this is the first study to assess the accuracy of the surface image guidance system across a complete 180° couch rotation.

Here a rigid phantom and a range of known positional offsets from isocenter were used to provide a cursory assessment of the accuracy of our surface imaging system in the absence of intrafraction motion. Our results show a mean deviation of 0.01 cm which is in accordance with phantom results from a previous study for which fixed, high‐precision couch‐mounted gauges were used.^9^

The added impact of intrafraction patient motion was addressed in volunteers. As expected, the use of volunteers increased the level of discrepancy between the rotated reference surface rendering centroid and the actual real‐time surface image of the volunteers. Bert et al reported a similar mean difference of 0.095 cm using partial breast irradiation patient population. As expected, the patient immobilization (i.e., thermoplastic mask) reduced the intrafraction motion (0.11 cm for nonimmobilized vs 0.08 cm for immobilized volunteers respectively). The magnitude of the observed setup deviations for the immobilized volunteers in this study are in agreement with what has been previously reported for surface imaging of the head and neck patient population.^4^

The recommended clinical action level to flag significant nonisocentric couch rotation is unique to our center and should not be implemented elsewhere without performing a local system assessment by a qualified medical physicist. In addition, most centers typically have a two‐pod surface imaging system installed, however, the work presented here was obtained from a three‐pod system (see Fig. 1). This study is limited in that it does not provide a direct comparison with the traditional two‐pod surface guidance systems found in most clinics. We expect some differences between the systems due to the reduced field‐of‐view.

The isocentricity and intrafraction motion was assessed using a surface rendering surrogate instead of the actual internal target. This is a limitation, since the position of the target is indirectly inferred through the position of an external surrogate. The validity of the assumption that the external surrogate position is correlated with internal target position has been studied in detail elsewhere.^12^ This study’s results were limited to healthy volunteers that did not receive x‐ray imaging, therefore a direct comparison between the surface and x‐ray image guidance was not presented.

Monitoring patient surface location is obviously not as precise a measure of target location as imaging of bony anatomy or fiducial markers. However, we believe it is a valid modality for a rough validation of a robotic rotation. Subtle runouts of 1–2 mm will not be identified with a 4‐mm action level, but systematic problems of that magnitude will be found with daily or monthly quality assurance. The goal of the surface image monitoring after radiographic localization is to identify large spurious errors that would have a clinically relevant impact on treatment quality. With the exception of some early couch motion problems that were associated with irregular workflows, no such error was identified after several years of radiographic validation of couch rotations, and so an argument could be made to suspend all such validation after the initial setup. Additionally, even in the absence of a couch rotation, by continuously monitoring the patient's surface image, we may identify intrafractional patient motion that should be corrected via radiographic imaging.

The system sensitivity and specificity was assessed for 2 and 4 mm clinical action levels. An analysis of the dosimetric impact of 2 and 4 mm positional deviations would be clinically beneficial; however, this was not provided and deemed outside the scope of this study. In our clinic, treatment plans are typically designed to be robust to setup uncertainties of up to 3–5 mm depending on the treatment site. Setting an action level of 4 mm on surface image deviation guards against spurious robotic errors that may be well in excess of our plan robustness — a phenomenon that has not been observed in years. Therapists are encouraged to perform subsequent x‐ray alignment checks if they have any reason to believe the target alignment has degraded beyond clinical tolerance, but they are not required to x ray after routine rotations. Additionally for SBRT cases where a single fraction variation of 3 mm in setup is significant, our practice is to continue to image with x rays between fields.

## 5 CONCLUSIONS

After characterizing the accuracy using phantoms and volunteers, we have shown that a three‐pod surface imaging system can be used to identify gross nonisocentric patient rotations. Significant positional deviations, either due to improper couch rotation or patient motion, should be followed by radiographic imaging and repositioning.

## CONFLICT OF INTEREST

None.
